# Intimate partner violence and abuse against Nigerian women resident in England, UK: a cross- sectional qualitative study

**DOI:** 10.1186/s12905-018-0610-4

**Published:** 2018-07-09

**Authors:** Omolade Femi-Ajao

**Affiliations:** Division of Dentistry, School of Medical Sciences, The University of Manchester, JR Moore Building, Manchester, UK

**Keywords:** Intimate partner abuse, Black women, Ethnic minority populations, Immigration status, Socialisation, Gender, Community groups, Faith-based Organisations, Acculturation, England, UK

## Abstract

**Background:**

Intimate partner violence and abuse is a public health problem affecting more than one third of all women globally. It usually takes place between individuals in intimate relationships and/or within the family. In the United Kingdom (UK), while theoretical and policy interventions have led to an increase in intimate partner violence and abuse service provision for women, there is paucity of research on the disclosure and help-seeking practices of women from ethnic minority populations.

**Methods:**

A cross-sectional qualitative research design was utilised. This included individual, in-depth semi-structured interviews with Nigerian women (*n = 16*) resident in England with lived experience of intimate partner violence and abuse. The interviews were conducted between May 2012 and May 2013, and data were analysed using thematic analysis technique.

**Results:**

Three main themes were identified as factors influencing the disclosure and help-seeking practices of Nigerian (ethnic minority population) women in England, UK. These are socialisation from country of birth, immigration status, and acculturation in the country of immigration.

**Conclusions:**

Nigerian (ethnic minority populations) women resident in England with lived experience of abuse are likely to seek help from leaders and members of their ethnic community groups and faith-based organisations. There is need for collaborative working with ethnic community groups and faith-based organisations to enhance access and facilitate the utilisation of existing intimate partner violence services.

## Background

Intimate partner violence and abuse (IPV) is a public health problem affecting more than one third of all women globally [1]. It usually takes place between individuals in intimate relationships and/or within the family [2]. Historically, little attention has been paid to the negative impact of intimate partner abuse [3, 4]. However, with the World Health Assembly in 1996 declaring violence against women a public health issue [5], and the resultant increase in evidence from empirical research highlighting the public health impact of IPV on women (eg: [6]), global regulations and conventions have been enacted to end violence and abuse against women and girls [7].

Globally, there are diverse statistics on the prevalence of IPV, which may reflect difficulties around disclosure and help-seeking. Estimates from a large WHO multi-country cross sectional survey of 24, 097 women aged 15–49 years showed that intimate partner abuse is widespread, and varied by cultures and countries [6, 8].

In the UK, existing evidence confirms that women are at a higher risk [9] of experiencing negative health impact from the abusive experience [5, 10, 11], and are likely to be killed by the abusive partner, which results in an average of two women being killed per week in England and Wales by a current or previous male partner [12].

Furthermore, evidence suggests that female victims of intimate partner abuse experience difficulty disclosing and help-seeking [13]. Thus, developments in the field of women’s health and gender studies over the last 20 years have led to renewed interest in improving support for female victims and survivors, with many support agencies and women’s rights activists group highlighting the need for governments and health care providers to enhance disclosure practices among women with lived experience of abuse [14, 15].

Although health care professionals may be in a position to help mitigate the short-term and long-term negative effects of abuse on women through early identification and intervention, especially when presenting with bruises and injuries in emergency care setting [16, 17], many of these women have been known to withhold information about their injuries [13].

Previous studies have reported that women, particularly women from ethnic minority populations, tend to distrust people outside their ethnic community, hence maybe reluctant to disclose their experience of abuse to persons unknown to them [18, 19]. Therefore, L Goodman, MA Dutton, K Weinfurt and S Cook [20] suggest that women with lived experiences of intimate partner abuse are likely to disclose such experiences to members of their informal networks, which may include family members, close friends and ethnic community group members, as opposed to health and social care professionals. Furthermore, M Kershner and JE Anderson [21] reported that women are likely to rely on self, God, family and friends in their disclosure of the abusive experiences. Thus, empirical evidence on disclosure emphasises the importance of understanding factors influencing the disclosure and help-seeking practices of women with lived experience of IPV, to aid the development of appropriate intervention and support strategies.

### Ethnic minority populations in the UK and intimate partner abuse

The past 30 years have seen increased diversity in the ethnicity of people living in the UK [22]. Although the White British ethnic group accounted for 80.5% of people resident in England and Wales in 2011, ethnic minority groups have increased from 6% in 1991 to 19.5% in 2011 [22]. While ethnic minority populations may also refer to migrant communities [23], they are often referred to as Black and Minority Ethnic (BME) groups in the UK.

According to the 2001 UK census definition, BME group is widely used to refer to individuals who belong to non-White British communities [24–26]. However, this term is not commonly used in other countries. For example, in Canada, ethnic minority groups are identified as migrant community [23]. Although the 2001 census’ definition refer to individuals who are not White-British, variants of the term ‘BME’ have been observed [27].

The 2001 census’ definition of BME is adopted in the paper, as it reflects a national recognition of the role of ethnicity and race on how persons from non-White British backgrounds identify themselves [24–26]. In addition, BME and ethnic minority populations are used interchangeably throughout this paper, in recognition of the varied nomenclature used to refer to immigrant communities, and as an attempt to foster consistency of terminology [27, 28].

In the UK, government policies and guidelines have led to initiatives such as *This Is Abuse* campaign [29] designed to raise awareness and provide support for victims and survivors. Despite these initiatives, women, especially from ethnic minority populations are less likely than White British women to disclose their experience of IPV to statutory services [30–32].

#### Ethnicity and IPV against women: Similarities and differences in the experience of British women and BME women

A large and growing body of literature have examined the impact of ethnicity on the experience of IPV among women in England [33–39]. I Law [40] refers to ethnicity as ‘the differentiation of groups of people who have shared cultural meanings, memories and descent, produced through social interaction’ (pg. 77).

Evidence from research on intimate partner violence among women in England has shown similarities and differences in the experience and impact of the abuse on women [35, 41, 42]. In a study conducted by J Hanmer [35], 30 British women and 30 BME women were asked about their experience of IPV. All women in the study identified similarities in how they understand violence, and the role of family in their experience. Themes such as feeling broken, blaming self, feeling depressed and severe emotional distress were common among all women. These commonalities have been found in another study by C Humphreys and R Thiara [41] in a study commissioned by Women’s Aid Federation, England.

However, there are differences in how women describe their experience of intimate partner violence and abuse. J Hanmer [35] found an inconsistent pattern of responding to IPV by staff from mainstream services (e.g. police, housing, social services) and unfavourable state policies where BME women are involved. J Hanmer [35] reported BME women as saying: ‘People say there’s this agency, that agency, and when it comes to us, there’s nothing’ (pg. 19). These findings support evidence from A Mama [37], and were reinforced by the work of J Batsleer, E Burman, K Chantler, SH McIntosh, K Pantling, S Smailes and S Warner [34], A Gill [43] and S Anitha [33]. In addition, E Burman, SL Smailes and K Chantler [44] asserts that, BME women face ‘the same obstacles in leaving violent relationships as white or cultural majority women – money, childcare, housing, transport; but each of these issues may also carry culturally specific inflections’ (pg. 336), which are exacerbated by racism, class position and immigration legislation [33, 44, 45].

Thus, while there are similarities in the incidence and type of IPV against women, there are differences in the response received by women from statutory services. The differences in the response from statutory services have been identified as exacerbating the negative impact of the abuse on BME women, as well as their disclosure and help-seeking practices [33]. The dynamics of BME women’s experience as a result of cultural inflections and problematization are also observed differences.

#### The Problematization of culture in IPV against BME women

Problematization is defined as a ‘strategy for developing a critical consciousness that disrupts taken-for-granted truths’ ([46], pg 1). Using the work of Foucault and others, Bacchi (2012) reports that ‘problematization involves a method of thinking, which examines how a phenomenon is questioned, analysed, classified and regulated, at specific times and in specific spaces’ (pg. 1). The way a phenomenon is problematized also influences policy and practice responses in tackling such a phenomenon [47, 48]. Thus, in the context of this paper, the phenomenon under investigation is the problematization of culture in IPV involving women from ethnic minority populations.

Evidence exists which suggest culture is problematized as a construct affecting only women from ethnic minority populations [39, 44, 47, 49–52]. E Burman, SL Smailes and K Chantler [44] suggests that culture discourse in the incidence of IPV against ethnic minority women is often critically examined and analysed (i.e. problematized) as either a homogenised absence or a pathologised presence. The homogenised absence/pathologised presence construct is a dichotomous representation, which on one hand excuses the incidence of IPV using generalised cultural reasons, thereby minimising the experience of ethnic minority women (homogenised absence), or overly scrutinising ethnic minority women with lived experience of IPV, thereby heightening their visibility (pathologised presence).

A core concept in this homogenised absence/pathologised presence dichotomy is culture blaming [44, 47, 50, 51]. Not only does culture blaming affect IPV service provision, it also significantly affects the utilisation of such services [42, 50]. Using the theoretical and conceptual definition of culture by D Matsumoto and L Juang [53], culture blaming is simplistic, since culture is dynamic and all societies have cultural values, beliefs, attitudes, and norms that influence their survival and existence [53, 54], and rather speculative, especially since it is often highlighted as being responsible for the incidence of abuse against ethnic minority women [34, 42, 44], and not white-British women [39].

Another challenged posed by the current way culture is problematized is that, it limits the struggles of BME women experiencing abuse to the margins [39], as it curtails their freedom to disclose and seek help for the abusive experience from mainstream services, for fear of heightened visibility or mis-representation of their culture or cultural orientation by the majority culture or host society [39, 52]. While there are harmful cultural practices which are acts of gender-based violence against women, the ecological model of understanding the experience of immigrant women experiencing IPV highlights the complexities in the interactions of women within and outside their cultures, and their host societies [55, 56].

Thus, it is important that the approaches to problematizing culture is expanded to accommodate other relevant consideration of the impact of culture and cultural orientation in the disclosure and help-seeking practices of women from ethnic minority population, with respect to help-seeking and IPV service utilisation. While there is a recognition within UK policy of the role of cultural orientation and the contribution of community groups and organisations to social welfare and public health [57, 58], the full potential of community groups and organisations have not necessarily been fully utilised, particularly around ensuring women from BME groups utilise existing IPV services.

This paper discusses the findings from a study conducted among Nigerian (ethnic minority population) women resident in the UK, who had experienced intimate violence and/or abuse from a current or previous intimate male partner, on factors influencing their disclosure and help-seeking practices.

## Methods

### Study design

A cross-sectional qualitative research design underpinned by interpretivist epistemological and ontological perspectives was utilised in this study. Thematic analysis technique by V Braun and V Clarke [59] facilitated the methodological approach used in data analysis.

### Study participants

We recruited 16 women who self-identified as Nigerian from Nigerian community groups and faith-based organisation and Manchester No Recourse to Public Funds (NRPF) team, using targeted and convenience sampling techniques [60–63]. Eligible participants were aged 18 to 54, and had a personal experience of IPV from a current or previous intimate male partner or husband.

### Researcher reflexivity

The important role of the researcher in qualitative research has been extensively studied [64]. Critics of qualitative research methodology constantly highlight the influence the researchers have on the research participants, and how this may interfere with the quality of the data generated [65]. Thus, being reflexive consists of the researcher’s awareness of her role in the research process and making conscious effort to ensure that her subjective role is minimised.

This was done by the researcher exploring and documenting her pre-conceived ideas, personal characteristics, epistemological and philosophical perspectives prior to the commencement of the qualitative study [66]. It was particularly important to ensure the researcher made a distinction between her voice, and the voice of the research participant, especially as this is an issue in interpretivist-based qualitative studies [67].

The researcher has certain characteristics which could be considered as placing her in a privileged position:

➢ Being Female

➢ Born and raised in Nigeria by married heterosexual parents

➢ Have a recognisable Nigerian (Yoruba) name

➢ Never experienced nor witnesses domestic violence and abuse (both as a child and adult)

The researcher’s first contact with someone who was a victim of IPV was in 2008 while working as a Graduate Research Assistant at a University in Nigeria. The student was very reluctant to speak about her experience. As the researcher had never experienced IPV, her first reaction was that of indignation, and further advice to the student to flee the relationship. The researcher was therefore very dismayed when this student married the perpetrator, saying he loved her and was angry with her for being bad. This was to be the beginning of the researcher’s interest in the decision-making process of seeking help for IPV by female victims and survivors.

Prior to commencing this study, the researcher had never met anyone who experienced IPV in England, but was aware of lack of published empirical research on IPV among Nigerian women in the UK, as she volunteered as a helpline worker in a Greater Manchester based domestic violence and abuse charity.

Her Nigerian upbringing was very instrumental in developing rapport and building professional relationship with the Nigerian group leaders. Thus, from the onset of this study, there were certain assumptions by the researcher and the study participants. An example of an assumption on the part of the researcher was that as all study participants were older in age than her. Due to this assumption, the researcher felt no need to ask study participants their age, particularly as the researcher is aware it was culturally unacceptable to ask your elders how old they were as a Nigerian. In addition, participants were not asked how many children they had, as these are not questions you were expected to ask people older than you, again due to its cultural unacceptability. However, during the course of the interview, some study participants felt confident to tell the researcher their age and whether or not they had children. This information was volunteered by the study participants, as part of discussing their experiences of living with IPV. Furthermore, the researcher perceived that study participants expected her to be respectful, by virtue of being a Yoruba girl and when they felt this expectation was met, the researcher posits this made the study participants confident and open about their participation in the study.

The use of reflexive journal and field notes were very useful in clarifying the researcher’s pre-conceived ideas, thereby ensuring a clear separation between her ideas and the data. One of the researcher’s assumption was that, study participants who were no longer in the abusive relationship must have made the firm decision to leave the perpetrator. This assumption was wrong, as half of the study participants who were no longer in the abusive relationship were abandoned by the male perpetrator.

Since the researcher and the researched are considered co-producers of knowledge within the interpretivist paradigm [68, 69], this had implication for data analysis. Thus, the researcher ensured that constant reference was made to the data extract, while ensuring her analytical interpretation of the findings were informed by her awareness of some of the cultural norms and practices within the Nigerian society in relation to IPV.

### Data collection

Following ethical approval from The University of Manchester Research Ethics committee, individual, in-depth, face-to-face and telephone, semi-structured interviews were conducted from May 2012 through May 2013 by the author [OF] in the homes of study participants, and at the University of Manchester, where safety of the participant and the researcher may be compromised. The interviews lasted for 90 min and were audio-taped. An interview guide was used to provide a focus for the interview, as well as set the boundaries for the research relationship between the research participant and the researcher [70].

The interview guide contained prompts, which allowed the researcher to link all the different sections of the topics, to ensure the research participant have the opportunity to provide quality data. Although the interview guide was handy to provide structure for the interview, research participants were allowed to tell their story, thus the researcher was not rigidly confined to the topics on the interview guide [70, 71]. As the interviews progressed, certain aspects of the interview guide were modified, as a result of allowing research participants to tell their story. Allowing the research participants to tell their story was a decision influenced by the research design, particularly the principles of the interpretivist paradigm.

### Data analysis

Each audio-taped interview was transcribed verbatim by OF and reviewed by a peer debriefer for credibility and dependability. In addition, a support team consisting of two experienced academics was convened that provided quality assurance and addressed the inter coder reliability throughout the data collection, transcription and analysis phases [72]. The transcribed interview data were explored using NVivo 10 software [73] to facilitate management, audit trail and organisation. The six phases of thematic analysis technique by Braun and Clarke (2006) was then implemented to generate relevant themes from the data. In keeping with the principles of anonymity, study participants were allocated pseudonyms. Table 1 below provides a descriptive summary of participants characteristics.

**Table 1:** Descriptive summary of Study Participants characteristics

S/N	Research ID (Pseudonyms)	Still in abusive relationship	Type of Abuse	Relationship with abusive male partner (marital status, where they met, and length of time together)	Have children	Affected by ‘No Recourse to public funds’ clause	Immigration status affected disclosure/help-seeking from Statutory services
1.	Aduke	No	Physical violence, controlling behaviour, financial abuse, sexual deprivation	Husband; met in Nigeria; 3 years	Yes	Yes	No
2.	Abike	No	Emotional abuse	Husband; met in Nigeria; 5 years	Yes	Yes	No
3.	Abeni	No	Physical violence, emotional abuse, sexual abuse, financial abuse	Partner; met in Nigeria; 2 years	Yes	Yes	Yes, overstayer^a^
4.	Abeke	No	Physical violence, emotional abuse, controlling behaviour, financial abuse	Husband; met in Denmark; 8 years	Yes	No	No
5.	Ajoke	No	Physical violence, emotional abuse, financial abuse, sexual abuse	Husband; met in Nigeria; 10 years	Yes	Yes	Yes, overstayer
6.	Anike	No	Physical violence, financial abuse, emotional abuse	Partner; met in England; 10 years	Yes	Yes	Yes, overstayer
7.	Agbeke	No	Physical violence	Husband; met in Nigeria; 5 years	Yes	Yes	Yes - overstayer
8.	Apinke	Yes	Physical violence, emotional abuse, financial abuse, sexual abuse, controlling behaviour	Husband; met in Nigeria; 20 years	Yes	Yes	Yes – dependent on his visa
9.	Akanke	Yes	Physical violence, emotional abuse	Husband; met in Nigeria; 12 years	Yes	Yes	No
10.	Amoke	No	Physical violence, emotional abuse. Financial abuse, controlling behaviour, sexual abuse (marital rape)	Husband; met in England; 5 years	Yes	No	No
11.	Asake	Yes	Physical violence, emotional abuse, sexual deprivation	Husband; met in England; 4 years	No	Yes	Yes - overstayer
12.	Asabi	No	Physical violence, emotional abuse	Husband; met in Nigeria; 23 years	Yes	Yes	Yes - overstayer
13.	Arike	Yes	Physical violence, emotional abuse, controlling behaviour	Partner; met in Nigeria; 37 years	Yes	No	No
14.	Alake	No	Emotional abuse, financial abuse, sexual deprivation	Husband; met in Nigeria; 10 years	Yes	Yes	Yes – dependent on his visa
15.	Amope	No	Emotional abuse	Husband; met in Nigeria; 7 years	Yes	Yes	Yes - overstayer
16.	Apeke	No	Physical abuse, emotional abuse, psychological abuse, controlling behaviour	Husband; met in Nigeria; 11 years	Yes	No	No

^a^In the UK, overstayer refers to a person who had stayed beyond the expiration of their visa

## Results

Three main themes were identified from the data. These are: socialisation from country of birth, immigration status, and acculturation in the country of immigration. The identified themes reflected the factors influencing disclosure and help-seeking practices of study participants.

### Socialisation from country of birth (Nigerian socialisation)

For participants in this study, socialisation from their country of origin was a key factor in their disclosure and help-seeking practices. Socialisation from country of origin determines the cultural norms, attitudes, behaviours, and it influences actions. Evidence from this study highlights the importance of Nigerian upbringing on the disclosure and help-seeking practices of women:

*I think what made me to stay for that long was because I wasn’t strong enough because of the society thing…. My strength would have been from what raised me, which is my culture. I didn’t have enough strength to say it’s all done, you know what, walk away. For me, the people I grew up with, my aunties, they endured it and their experiences were really very bad, and they are still in it, and this was what was modelled to me. So basically, what was modelled to me was what made me stay, I couldn’t file for divorce* **(Abike)**.

*You know we Nigerian women, the way we were brought up, you know in your house they will tell you, in your husband’s house, this is what will happen, you don’t have to complain* (**Agbeke**)

*You know what, based on our culture of suffering and smiling, this prevents many of us from speaking up and seeking help…. And what will people say? That you are telling others about your man, such things tie us up, and it’s the cause of our problem (* ***Amope*** *)*

Nigerian women said they actively disclosed to and sought help from persons considered to be in positions of authority within the Nigerian ethnic group, as a way of seeking help for the IPV experience, while upholding their family unit.

*So, the first person I really opened up to was Chief* [community leader], *she listens to me, and she doesn’t say anything bad, she just listens and did not tell other people my story* **(Arike)**

*The first person I spoke with was a member of the church, like an elder. And she asked me to speak with the pastors. So I spoke with the pastor. I think they were the first before I ever spoke with my parents* **(Alake)**.

*When the abuse became unbearable, I spoke with his elder sister first. She said, I have to endure the hardship, and that in this country, the woman does more than the man. That is, I have to do more to support the family. My ex-partner will do 30% and I will do 70%* (**Anike**)

This act of actively seeking help from Nigerian ethnic group leaders and respected family members has a significant influence on the disclosure and help-seeking practices of study participants.

#### Immigration status

Fifteen of the 16 study participants were first generation immigrants from Nigeria, who came to the UK on the entry or residence permit (visa) of their partner or husband. Due to the condition of their immigration/residence permit, study participants were not entitled to welfare support if their relationship breaks down. Findings from this study showed that participants were likely to endure the abuse for fear of visibility, destitution, and deportation:

*I was scared really because of our status. Our status was one of the main reasons for not speaking. I’ve been told, and my husband also told me, that listen, if you continue to go to the police when I do things to you, we are both going to be deported...* **(Ajoke)**

*…anytime we have any slight argument,* [the abusive husband says] *I’m moving out, I’m moving out. Even recently, he* [abusive husband] *was going to move out, and I had to ask people to beg, because of my financial status and my immigration issue, so I had to ask people to beg him.* **(Asake)**

*…and you know the physical violence was going on, and he was like if I threaten him with the police, he will threaten me with taking us back home. I just looked at it, number one, I am not entitled to anything, if I reported him, it will worsen the whole situation, so I just kept keeping it within me…* (Apinke)

*I told one of our nurse managers, and she gave me numbers that I could call, like women’s aid, social services, and children social services to tell them. I collected the number, but I didn’t do anything, I kept quiet, because I didn’t have papers*^1^ (**Asabi**).

*…the fact that I was in the UK as a dependent stopped me from telling anybody, I will say the white people. I know that if anything goes wrong, then I will be going back with my two children (* ***Alake*** *)*

#### Acculturation in the country of immigration

As mainly first-generation immigrants, study participants described the process of acculturation in their adaptation to the culture in England. Acculturation is ‘defined as a culture learning process experienced by individuals who are exposed to a new culture or ethnic group’ ([74]: pg 102). Study participants disclosed that their acculturation in England determined whom they trusted and associated with.

While study participants were mostly educated from Nigeria, and have a level of proficiency in English language prior to coming to England, it was their experience that the acculturation process was prolonged for them, particularly with regards to securing employment, developing friendships and learning the culture in England:

*I came down here heavily accented, I couldn’t take on paid employment…* **(Apinke)**

*I went to Women’s Aid and explained my problems, told them I don’t have anywhere and my husband wants to kill me. When I explained my problems, they said to me, why can’t you call the police? I couldn’t answer the question properly because it’s not my language and as I am new in England, I don’t know how they do here* (**Aduke**)

In addition, it was the experience of some study participants that they were abandoned by their abusive male partners in England and were left with no means of sustenance. Being abandoned exacerbated their distressed caused by the IPV experience, especially due to immigration fears and limited financial resources.

*…he* [husband] *stopped contributing to rent. Before that time, he was not living in the house most of the time. When he stopped contributing to the rent, he told the landlord I should be the one paying the full rent which I couldn’t afford at the time. But it became very bad and I had to move out of the house to a one bedroom flat with my children. After a year, he filed for divorce that I had moved out of the house* **(Abike)**

*He left me and the children here with nothing and went back to Nigeria. He did not want to be here, knowing that, when he does something to me, there are people that will stand up for me, he couldn’t stand that, and that was his reason for going back* (**Ajoke**).

Although the impact of acculturation has been linked to economic pressures, and stress of integrating to the new cultures [75], it has not been discussed in terms of negatively affecting help-seeking from statutory services, especially as the rationale for lack of confidence and trust in seeking help. With respect to seeking help for IPV, findings from this study point to acculturation as being influential in whether a woman will disclose to and seek help from statutory services, particularly on issues of trust and confidentiality. The lack of trust and confidence in utilising statutory services has been critically discussed based on how the culture of ethnic minority women is problematized [34, 44, 49, 50].

## Discussion

The aim of this study was to identify factors influencing the disclosure and help-seeking practices of Nigerian (ethnic minority population) women resident in England with lived experience of intimate partner violence and abuse. Three main factors which are influenced by values of familism, trust and confidentiality were identified in this study.

### Values of familism

Socialisation from country of origin as a factor influencing the disclosure and help-seeking practices of Nigerian women in England corroborates findings from the work of UA Kelly [18] among Mexican-Americans. Using evidence from her work with Latino immigrant women, UA Kelly [18] underscores the role of family, care of children and socialisation in the disclosure and help-seeking practices of women. Thus, it could be deduced that the familism values prevalent in the Latino literature (for example: [76–82]) are similar to the values described by Nigerian women as influencing their disclosure and help-seeking practices.

By definition, the term familism:

*‘…refers to a model of social organization, based on the prevalence of the family group and its well-being placed against the interests and necessities of each one of its members. It is part of a traditional view of society that highlights loyalty, trust and cooperative attitudes within the family group…’* ([83]: pg 546).

Theoretically and conceptually, familism refers to upholding the goals of the family as premier rather than the individual family members’ needs, and expectation that the family unit will support its members [56, 80, 83–85]. While familism has been criticised for its dominance and controlling tendencies towards its family members [83, 84], it has been positively linked to improved mental health, strong social support networks, and acculturation within immigrant communities in the United States [78–80, 82].

L Grebler, J Moore and R Guzman [81] maintains that while there is a distinction between the manifestation of familism within traditional and modern families, the social model of familism is still evident in both nuclear and extended families. This position has been supported by A Garzon [83], as she argued that despite the changes in family structure, (for example, change from the traditional view of family as comprising of heterosexual father as male and mother as female), increase in divorce rates, and views about children, familism still influences societal attitudes, beliefs and values.

The values of familism has been suggested as serving to protect, support and strengthen the members of the family [78, 82]. Espousing familism as important in the socialisation of the child and its later influences on decision-making is necessary to understand the practices of Nigerian women with lived experience of IPV in England. Within the Nigerian literature, discourses of familism are not prevalent. Although B Oloko [86] through her study on child socialisation in urban Nigeria establishes that familism values is the core of the social structure and development of the Nigerian child, as children are thought the values of respect for and commitment to the family unit, discussions regarding the role of familism are usually explored from patriarchal perspectives, which may be detrimental to the structure and function of the family, particularly in view of changes in the definition and shift in the concept of family [83].

Using patriarchal perspectives to explain socialisation and familism is also detrimental in that it tends to present the family along traditional gendered roles of women as subordinates that are controlled by men. While this may be true in certain instances, evidence from this study suggests Nigerian women in England still uphold the values of familism, and defer to their extended families in Nigeria despite residing in England, an individualistic society with less emphasis on collectivist cultural orientation [87, 88].

#### Trust and confidentiality

The importance of trust and confidentiality in disclosure and help-seeking for IPV has been established in literature [89, 90]. In her literature review, M Barnish [89] highlighted that lack of trust in social services and the judicial system was a significant barrier to utilising such services. For study participants, who were socialised within the collectivist society of Nigeria, and were not previously accustomed to utilising statutory services for IPV in their home country, trust and confidentiality was very important. Hence, this was reflected in their disclosure and help-seeking practices.

Rather than utilise statutory services to help with their abusive experiences, Nigerian women were likely to seek confidential help from members of their ethnic community groups. This is similar to findings from the work of AM Akinsulure-Smith, T Chu, E Keatley and A Rasmussen [91] on intimate partner violence among West African immigrants in the USA. Seeking help primarily from Nigerian ethnic group leaders was identified as having a tendency to negatively impact on statutory services responses to Nigerian women who self-refer for help. This is due to issues regarding the documentation of the incidence of the abuse, as Nigerian women may not have previously logged the abuse with the police, their GPs or other services in a position to provide documentary evidence in support of the incidence of the abuse [92].

#### Link between familism and acculturation

Acculturation may be linked to familism, in the case of participants in this study. Evidence from this study indicates that in the absence of extended family in England, Nigerian women developed religious kinship. The process of developing religious kinship has been described as religious familism by P Edgell and D Docka [93]. In their study on gender ideology and familism in religious communities, P Edgell and D Docka [93] found that in religious communities where familism values are upheld, women’s well-being issues such as IPV are treated with great importance. Therefore, it may be suggested that Nigerian women seeking help for IPV from religious leaders perceived such religious congregations as family.

This notion may also be extended to community familism, as a result of evidence from this study. It also suggests that adherence to the values of familism is inherent in the social learning of Nigerian women. Thus, interventions designed to enhance disclosure and help-seeking, as well as service utilisation to protect the health and well-being of women needs to be oriented towards integrating family values.

### Developing a disclosure and help-seeking model

Study participants who were first generation immigrants, with no recourse to public funds and living within Britain’s individualistic society, recounted difficulties acclimatising to life in Britain due to a variety of reasons, and were unaware of sources of support for their IPV experience outside their Nigerian religious or community groups. Hence, it was suggested by study participants that an organisation be established to facilitate access to statutory and other specialist services. As indicated earlier, similar recommendations have been made by B Liang, L Goodman, P Tummala-Narra and S Weintraub [94] to help immigrant women navigate statutory services in their country of immigration.

**Fig. 1:**
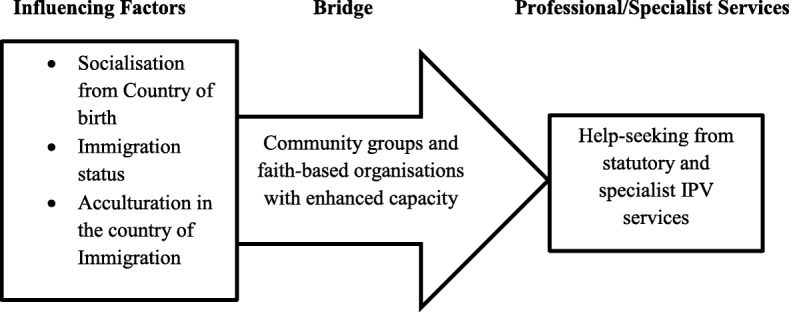
Disclosure and Help-seeking Model to Enhance Service Utilisation

B Liang, L Goodman, P Tummala-Narra and S Weintraub [94] developed a theoretical model of help-seeking for IPV, which considered the influences of individual, sociocultural and interpersonal characteristics on help-seeking decision and support selection. In addition to data from this study, evidence from B Liang, L Goodman, P Tummala-Narra and S Weintraub [94] was useful in developing the help-seeking model presented in Fig. 1, which may enhance the utilisation of statutory and other specialist services for IPV by individuals from ethnic minority populations resident in the UK.

### A case for collaborative working with community groups and faith-based organisation in IPV prevention and intervention approaches in the UK

Evidence from the review and synthesis of literature, particularly from the US have shown that ethnic community groups (CGs) and faith-based organisations (FBOs) are avenues for research participation, implementing health interventions and outreach programs, and for disseminating health information [95–97]. Findings from this research have highlighted the role of community groups and faith-based organisations in supporting study participants. Therefore, it is the suggestion of this paper that in the UK, community groups and faith-based organisations could be avenues for accessing and supporting women (and men) from ethnic minority populations with lived experience of abuse, in order to facilitate their utilisation of existing IPV services. Similar suggestions have been made based on research among Korean Americans, Mexican Americans, Asian Americans, and Immigrants communities in Canada [4, 94, 98–101].

Community groups and faith-based organisations have been recognised as important drivers in social development and sustainability of interventions designed to improve health and wellness [57, 102–105]. The UN System [106] entities and specialised agencies have been observed to utilise CGs and FBOs, particularly in developing countries, to increase the uptake of childhood immunisations, combat violence, support internally displaced persons, as well as implement health education and promotion programs and interventions [107–109].

In the UK, community groups and faith-based organisations have been recognised as contributing significantly to improving the social welfare of the public [104, 105, 110]. This recognition has been further enhanced due to the inclusion of religion and belief in the Equality Act 2010 [57], and has led to increased funding opportunities for community groups and faith-based organisations to provide certain publicly funded services [111]. However, in recent times, CGs and FBOs have been lumped together with all other non- governmental organisations under the broad category of third (voluntary and community) sector organisation. While this may be advantageous in that it promotes equality and diversity, it has a tendency to undermine the extensive social capital resources existing within CGs and FBOs [105].

RD Putnam [112] refers to social capital as the ‘features of social life – networks, norms, and trust – that enable participants to act together more effectively to pursue shared objectives’ (pg. 664) and suggested that ‘the theory of social capital presume that, generally speaking, the more we connect with other people, the more we trust them’ (pg. 665). Therefore, trust is a significant component of social capital, and influences the social structure within which communities and groups exists [105]. The work of C Hepworth and S Stitt [105] on social capital and faith-based organisations suggested approaches for enhancing the capacity of FBOs to ensure active involvement in community development.

As observed in this study, trust was very important in the disclosure and help-seeking practices. Study participants strongly suggested the establishment of an organisation to enhance their access to statutory and mainstream IPV services. This suggestion might have been influenced by the existing social capital within their UK social structure networks. However, establishing a new organisation might prove difficult for a number of reasons, such as availability of funding, risk assessment, and management structures of the organisation, among others. Thus, rather than establish a new organisation, there are existing governmental agencies whose remit may be extended to incorporate community groups and faith-based organisations in promoting intimate partner violence and abuse prevention and intervention activities. One of such organisations is Public Health England.

### Collaborative work with Public Health England

Public Health England (PHE) is an executive agency of the Department of Health established in 2013 to ‘protect and improve the nation’s health and wellbeing, and reduce health inequalities ([113], pg 1). Due to the structure and framework of Public Health England [114], it has the resources and manpower to involve CGs and FBOs as part of plans to directly support the public to improve their health and wellbeing [114], particularly with respect to making IPV information available to ethnic minority populations, and also coordinating the training and capacity building of leaders of CGs and FBOs to enable them appropriately support individuals experiencing intimate partner abuse (see Fig. 1).

Involving community groups and faith-based organisation as part of the remit of PHE is a community approach to health and wellbeing which could be challenging. This type of partnership approach, which involves working with community groups and faith-based organisations to enhance health and wellbeing has been explained using the complexity theory [115].

SB Yosef [115] asserts that:

‘*The community approach enables professionals from varied disciplines (police officers, doctors, social workers, community center directors, urban planners, attorneys, etc.) to deal with social challenges while making use of both their professional expertise and the community approach. As the awareness of the necessity of partnerships grows it becomes more and more complicated to apply it. The complexity theory provides a guideline for handling that challenge successfully*’ (pg.1).

Although complexity theory is an emerging science and a combination of concepts, it has been described as a framework for understanding all aspects of human interactions and behaviour, and has been identified as necessary for fostering new approaches to child protection, healthcare management, translating evidence into practice, and community approach to development [116–118]. The complexity theory framework includes concepts such as self-organisation, emergence, and non-linear understanding [103, 118, 119] to enable practitioners work together to find sustainable solutions to existing problems, and foster understanding of the differences in their organisational compositions and approaches to problem-solving.

Thus, when applied to involving CGs and FBOs as avenues for ensuring the utilisation of existing IPV services (including prevention and post-separation services), the complexity theory will enable CGs, FBOs, and Public Health England to work within a framework that recognises the strengths of each group and create an enabling environment that foster understanding and sustainable approaches to protecting and improving the health of persons from ethnic minority populations affected by IPV. By working collaboratively with Public Health England, ethnic CGs and FBOs would possess enhanced capacity to support women subject to immigration control, and/or those who have stayed beyond the expiration of their visas, thereby ensuring women are in a better position to make informed decision regarding help-seeking for their IPV experiences. Furthermore, this will contribute towards the evidence base required for gender-sensitive polices focused on improving the health status of ethnic minority populations.

#### Immigration context

Although there is plethora of evidence on the use of immigration status and threat of deportation by the male abusive partner [4, 19, 43, 85, 120], particularly where only the woman is subject to immigration control, little is known about how immigration status affect disclosure and help-seeking practices where both the abusive male partner and the woman are subject to immigration control. Thus, the role of immigration status in the context of IPV involving women from ethnic minority populations, where both the abusive male partner and the female victim were subject to immigration control was identified as an original contribution of this study.

### Strengths and limitation

The strength of this study is that, it was successful in achieving its stated aim and objectives. Evidence from this study highlighted the perspectives of Nigerian women in England experiencing IPV. As a study focused predominantly on Nigerian (Black African) women, it adds to existing research on the disclosure and help-seeking practices for IPV by women from ethnic minority populations in the UK.

However, a number of limitations need to be considered. First limitation lies in the fact that, a significant number of study participants were recruited from Nigerian religious and ethnic community groups. This has implication for how the findings are interpreted, as it excludes the views of women who do not practice any religion or participate in Nigerian community group functions.

Secondly, study participants were mainly first generation economic immigrants who came to Britain as dependents of work permit holders, MSc and PhD students, or holders of visitor’s visas. Hence, this study sample is not representative of the Nigeria population in England [121], as it does not include Nigerians who are asylum seekers or refugees. As the views presented in this paper are from the perspectives of people who possess to a large extent some level of socio-economic status, caution must be applied, as the findings might not to be transferable to all Nigerian women with insecure immigration status. Thus, there is a challenge to ensure appropriate representation of views, in consideration of the multifaceted nature of intimate partner abuse.

## Conclusion

The original contribution of this paper to knowledge is that it highlights the role of immigration status in the disclosure and help-seeking process where both the male abusive partner and the abused women are subject to immigration control. It also contributes to existing discourse on the problematization of culture, by highlighting the important roles of community groups and faith-based organisations in health promotion, health education, research, and intervention [58]. Specifically, findings from this research support previous evidence advocating for collaborative working with ethnic minority community groups and faith-based organisations, in order to enhance positive health behaviour, reduce health inequality and facilitate the utilisation of existing services among ethnic minority populations [58, 96, 97, 100, 122].

## Abbreviations

BME: Black and Minority Ethnic
CG: Community group
FBO: Faith-based organisation
IPV: Intimate partner violence and abuse
NRPF: No recourse to public funds
PHE: Public Health England
UK: United Kingdom
UN: United Nations
US: United States of America
WHO: World Health Organisation

## Footnotes

1 papers refer to legal immigration documents
